# Comparisons of auditory brainstem response and sound level tolerance in tinnitus ears and non-tinnitus ears in unilateral tinnitus patients with normal audiograms

**DOI:** 10.1371/journal.pone.0189157

**Published:** 2017-12-18

**Authors:** Hyun Joon Shim, Yong-Hwi An, Dong Hyun Kim, Ji Eun Yoon, Ji Hyang Yoon

**Affiliations:** Department of Otorhinolaryngology-Head and Neck Surgery, Eulji University School of Medicine, Eulji Medical Center, Seoul, Korea; Universidad de Salamanca, SPAIN

## Abstract

**Objective:**

Recently, “hidden hearing loss” with cochlear synaptopathy has been suggested as a potential pathophysiology of tinnitus in individuals with a normal hearing threshold. Several studies have demonstrated that subjects with tinnitus and normal audiograms show significantly reduced auditory brainstem response (ABR) wave I amplitudes compared with control subjects, but normal wave V amplitudes, suggesting increased central auditory gain. We aimed to reconfirm the “hidden hearing loss” theory through a within-subject comparison of wave I and wave V amplitudes and uncomfortable loudness level (UCL), which might be decreased with increased central gain, in tinnitus ears (TEs) and non-tinnitus ears (NTEs).

**Subjects and methods:**

Human subjects included 43 unilateral tinnitus patients (19 males, 24 females) with normal and symmetric hearing thresholds and 18 control subjects with normal audiograms. The amplitudes of wave I and V from the peak to the following trough were measured twice at 90 dB nHL and we separately assessed UCLs at 500 Hz and 3000 Hz pure tones in each TE and NTE.

**Results:**

The within-subject comparison between TEs and NTEs showed no significant differences in wave I and wave V amplitude, or wave V/I ratio in both the male and female groups. Individual data revealed increased V/I amplitude ratios > mean + 2 SD in 3 TEs, but not in any control ears. We found no significant differences in UCL at 500 Hz or 3000 Hz between the TEs and NTEs, but the UCLs of both TEs and NTEs were lower than those of the control ears.

**Conclusions:**

Our ABR data do not represent meaningful evidence supporting the hypothesis of cochlear synaptopathy with increased central gain in tinnitus subjects with normal audiograms. However, reduced sound level tolerance in both TEs and NTEs might reflect increased central gain consequent on hidden synaptopathy that was subsequently balanced between the ears by lateral olivocochlear efferents.

## Introduction

Recently, “hidden hearing loss” with cochlear synaptopathy has been suggested as a potential pathophysiology of tinnitus in patients with normal hearing thresholds. Kujawa and Liberman observed a permanent loss of ribbon synapses and auditory nerve fibers in the high frequency region in mice after recovery of hearing thresholds following acoustic trauma. This finding is well correlated with the reduction in wave I amplitude in auditory brainstem responses (ABRs) after similar recovery of hearing thresholds [1]. In several human studies, participants with tinnitus and normal audiograms showed significantly reduced ABR wave I amplitudes compared with control subjects but exhibited recovery of wave V amplitude to normal levels [2,3]. The authors hypothesized that selective deafferentation of high-threshold auditory nerve fibers induced tinnitus, whereas low-threshold fibers were intact, thus preserving the hearing threshold [2,4,5]. Similarly, tinnitus patients with normal audiograms exhibit increased tone-detection thresholds for threshold-equalizing noise at high sound intensities [6]. Pienkowski and Eggermont exposed cats to an enhanced acoustic environment (6-week sound exposure level of 68 dB SPL) and found reorganization of the primary auditory cortex tonotopic map without a shift in auditory brainstem response thresholds [7]. This result implies that neuronal plasticity in the auditory cortex could be triggered by changes in peripheral input that do not impact hearing threshold. Therefore, normal hearing thresholds do not necessarily reflect an absence of damage to the cochlea or auditory nerve.

According to the theory of “hidden hearing loss”, the potential of wave V increases as a compensatory response to reduced wave I potential, thus increasing excitatory gain in the central auditory system. This homeostatic plasticity in response to decreased input signal is a potential mechanism of tinnitus. Reduced signal transduction due to damaged cochlear hair cells, ribbon synapses, or a damaged cochlear nerve may reduce lateral inhibition and thus increase spontaneous activity, or may induce synchronous firing of the central auditory tract or auditory cortex, i.e., “maladaptive neural plasticity” [8,9,10].

Comparative analyses of the wave amplitude of ABRs should consider the way in which wave amplitude varies with factors such as age, sex, and the physical properties of the head. Within each sex, there is large variation in individual measurements of amplitude, and amplitude varies more between subjects than within subjects [11]. Thus, within-subject comparison of wave amplitude could be more reliable than previous between-subjects comparisons (tinnitus group vs. control group).

In the current study, we aimed to reconfirm the “hidden hearing loss” theory through a within-subject comparison of tinnitus ears (TEs) and non-tinnitus ears (NTEs) with respect to wave I and wave V amplitude. In addition, we evaluated the difference between TEs and NTEs for uncomfortable loudness level (UCL) as a potential factor reflecting the increased central auditory gain. If “hidden hearing loss” with cochlear synaptopathy were a main pathophysiology of tinnitus in subjects with normal audiograms, we would expect these subjects to exhibit decreased UCLs due to over-amplification of input signal.

## Materials and methods

### Subjects

Human subjects included 43 unilateral tinnitus patients (19 males: 28.58 ± 10.88 years, 24 females: 37.58 ± 14.38 years) with normal and symmetric hearing thresholds (≤ 20 dB HL at 0.25, 0.5, 1, 2, 3, 4, and 8 kHz and a binaural difference of ≤ 10 dB at all measured frequencies).

The mean duration of tinnitus was 7.61 ± 16.66 and 7.15 ± 13.25 months in the male and female groups, respectively. More information about the participant tinnitus characteristics is listed in Table 1.

**Table 1:** Characteristics of tinnitus in tinnitus subjects.

Male tinnitus subjects					
Pitch (kHz)	Duration (month)	THI score	VAS of the loudness	^a^ TAS (%)	BDI
5.13 ± 2.26	7.61 ± 16.66	45.37 ± 26.89	4.79 ± 1.84	64.21 ± 37.02	13.68 ± 7.67
Female tinnitus subjects					
Pitch (kHz)	Duration (month)	THI score	VAS of the loudness	^a^ TAS (%)	BDI
3.07 ± 3.29	7.15 ± 13.25	44.79 ± 26.48	5.46 ± 2.23	55.83 ± 32.83	12.57 ± 7.77

THI, tinnitus handicap inventory; VAS, visual analogue scale; BDI, Beck depression inventory

^a^Tinnitus awareness score (TAS) is defined as the percentage of time during which the patient is aware of tinnitus in one day.

We also enrolled 18 age- and sex-matched control subjects (8 males: 28.50 ± 6.97, 10 females: 28.70 ± 11.7810) with normal and symmetric hearing thresholds (same criteria used for the tinnitus group). Table 2 shows the mean pure-tone averages for tinnitus and normal subjects. Both ears from each member of this group were used as control ears.

**Table 2:** Age, sex, and pure-tone average (PTA) for tinnitus and normal subjects.

	Tinnitus subjects (n = 43)				Normal subjects (n = 18)			
	Male (n = 19)		Female (n = 24)		Male (n = 8)		Female (n = 10)	
Age (years)	28.58 ± 10.88		37.58 ± 14.38		28.50 ± 6.97		28.70 ± 11.78	
PTA (dB hearing level)	TE	NTE	TE	NTE	Right	Left	Right	Left
	5.64 ± 2.63	4.79 ± 2.51	8.85 ± 5.21	7.44 ± 4.16	8.56 ± 3.04	7.16 ± 5.13	6.70 ± 2.83	5.85 ± 3.13

PTA, pure-tone average; TE, tinnitus ear; NTE, non-tinnitus ear

All subjects underwent audiological tests, including pure-tone audiometry, tinnitus pitch matching, impedance audiometry, and ABR. After careful physical examination of the head and neck regions of each participant, we excluded those suspected of having objective tinnitus or somatic tinnitus from the study. Subjects with chronic otitis media, retrocochlear lesions, endolymphatic hydrops, or congenital ear malformation were also excluded. Finally, we excluded subjects with tinnitus accompanied by dizziness, a history of ototoxic drug use. This study was approved by the Institutional Review Board of the Eulji Medical Center, South Korea and all participants provided their written informed consent.

### Procedure

We used a Navigator Pro Auditory Evoked Potential system (Bio-logic, New York, USA) to measure ABRs and insertable earphones (Etymotic, ER-3A) to present click sounds. We measured the amplitudes of wave I and V from the peak to the following trough and the latencies of wave I and wave V two times each at 90 dB normal hearing level (nHL, 13.3 per sec click, 1500 sweeps) and used the average data from two trials (S1 Fig). The noise level was kept at zero during the measurement process, and the sweep number was standardized at 1500. We conducted psychoacoustic measurements of UCLs with 500 Hz and 3000 Hz pure tones in the TE and NTE groups separately. The stimuli were routed through an audiometer (AC40; Interacoustics, Middelfart, Denmark) and presented monaurally to each test ear via headphones (TDH-39P; Telephonics, New York, USA).

### Analysis

Because of known sex difference in wave amplitude, we analyzed ABR data separately according to sex. We compared the average amplitudes of wave I, wave V, and the wave V/I amplitude ratio, and the average latencies of wave I, wave V, and the wave I-V interval for the TEs and NTEs in each male and female tinnitus group using a paired *t* test or Wilcoxon test. We compared average UCLs at 500 Hz and 3000 Hz for the TEs and NTEs of all tinnitus subjects (without sub-grouping according to sex) using a Wilcoxon test. Statistical comparisons of the amplitude and latency of wave I, wave V, and UCLs among the TEs, NTEs, and control ears in normal subjects were performed using multiple independent *t* tests or Mann-Whitney tests with the Bonferroni correction (α = 0.05/3 = 0.017). We analyzed the correlations between wave I and V amplitudes in the TEs, NTEs, and control ears via Pearson’s correlation analysis. Additionally, for each of the UCLs at 500 Hz and 3000 Hz in TEs, we analyzed correlations between a) the wave V/I amplitude ratio and b) the wave I amplitude using Spearman’s correlation analysis.

## Results

### Comparisons of amplitude and latency of wave I and wave V for TEs and NTEs in male subjects with unilateral tinnitus

**Fig 1:**
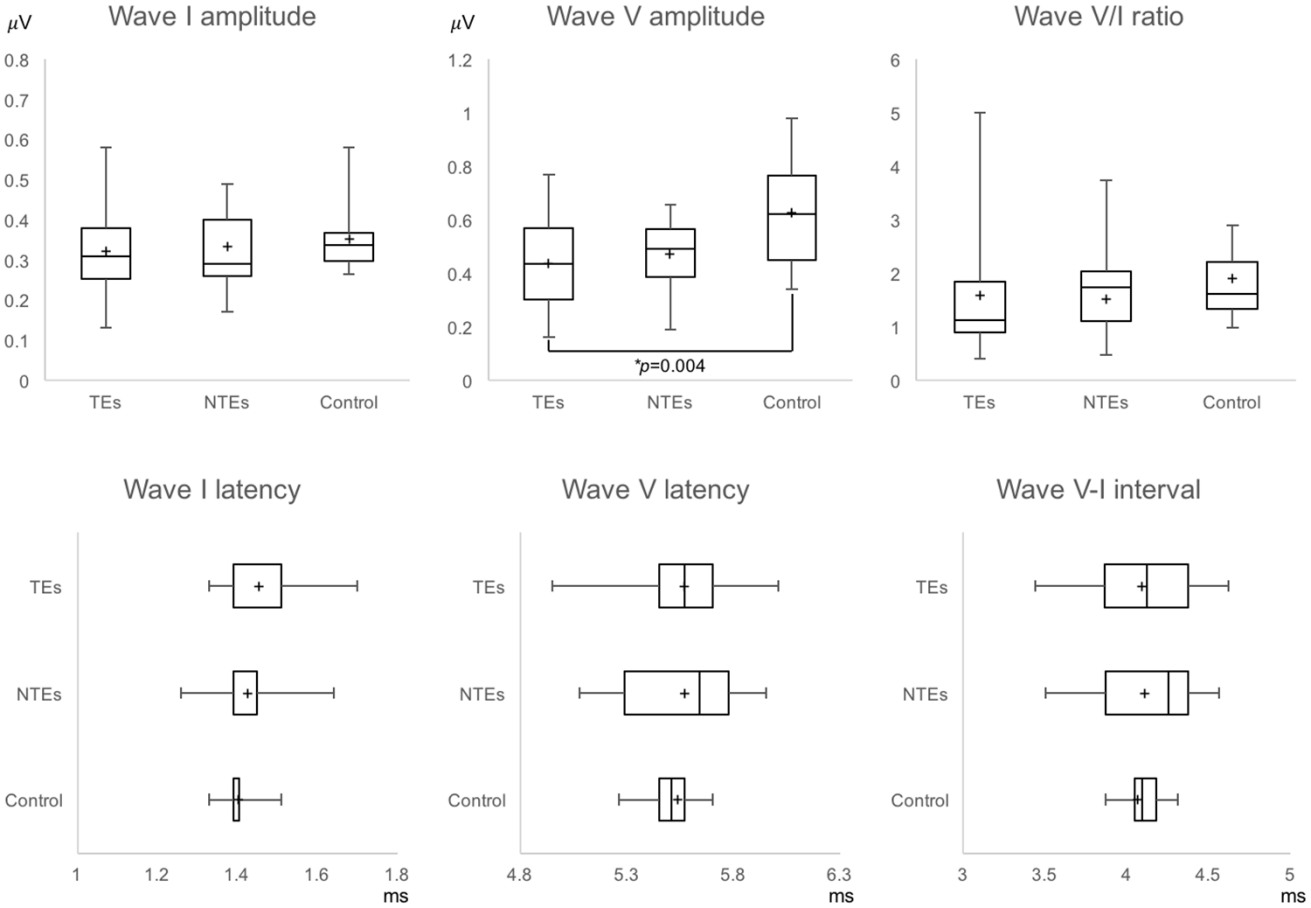
Comparisons of wave I and wave V among the TEs, NTEs, and control ears in male subjects with unilateral tinnitus. Each box indicates the 25th to 75th percentile range and whiskers indicate minimum and maximum values. Vertical bars indicate the median, + indicates the mean. TEs, Tinnitus ears; NTEs, non-tinnitus ears, * *p* < 0.05.

The within-subject comparison between TEs and NTEs in the male tinnitus group revealed no significant differences in the amplitudes of wave I and wave V, or in the wave V/I amplitude ratio (paired *t* test or Wilcoxon test, *p* > 0.05). Similarly, multiple comparisons among the TEs, NTEs, and control ears in normal male subjects with the Bonferroni correction revealed no significant differences in the amplitudes of wave I and wave V, or the wave V/I amplitude ratio (independent *t* test or Mann-Whitney test with the Bonferroni correction, *p* > 0.017; α = 0.05/3, Fig 1), with the exception that the amplitude of wave V was lower in the TEs vs. control ears (Mann-Whitney test with the Bonferroni correction, *p* = 0.004; α = 0.05/3, Fig 1). Our latency comparison revealed no significant differences in the latencies of wave I and V, or the wave I-V interval between TEs and NTEs (Wilcoxon test, *p* > 0.051). Multiple comparisons of latency also revealed no significant differences among any groups, including control ears (independent *t* test or Mann-Whitney test with the Bonferroni correction, *p* > 0.017, Fig 1).

### Comparisons of amplitude and latency of wave I and wave V for TEs and NTEs in female subjects with unilateral tinnitus

**Fig 2:**
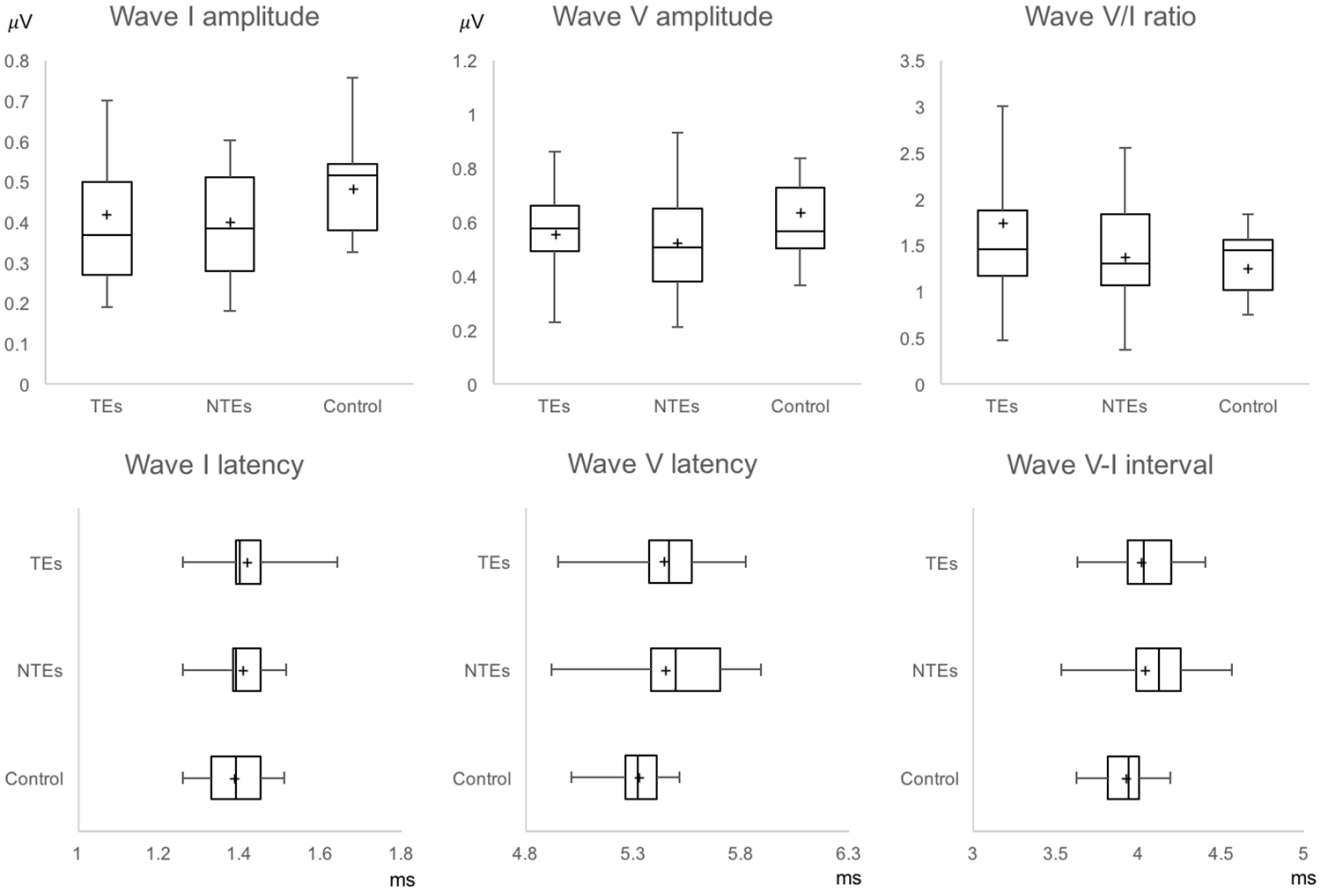
Comparisons of wave I and wave V among the TEs, NTEs, and control ears in female subjects with unilateral tinnitus. Each box indicates the 25th to 75th percentile range and whiskers indicate minimum and maximum values. Vertical bars indicate the median, + indicates the mean. TEs, Tinnitus ears; NTEs, non-tinnitus ears.

The within-subject comparison between TEs and NTEs in the female tinnitus group revealed no significant differences in the amplitudes of wave I and wave V, or the wave V/I amplitude ratio (paired *t* test, *p* > 0.05). Multiple comparisons among the TEs, NTEs, and control ears in normal female subjects with the Bonferroni correction revealed no significant differences in the amplitudes of wave I and wave V, or the wave V/I amplitude ratio (independent *t* test or Mann-Whitney test with the Bonferoni correction, *p* > 0.017; α = 0.05/3, Fig 2). We also found no significant differences in the latencies of wave I and V, or the wave I-V internal between TEs and NTEs (paired *t* test or Wilcoxon test, *p* > 0.05). Multiple comparisons of latency also revealed no significant differences among any groups, including control ears (independent *t* test or Mann-Whitney test with the Bonferoni correction, *p* > 0.017, Fig 2).

### Comparisons of UCLs at 500 Hz and 3000 Hz for TEs and NTEs in all subjects with unilateral tinnitus

**Fig 3:**
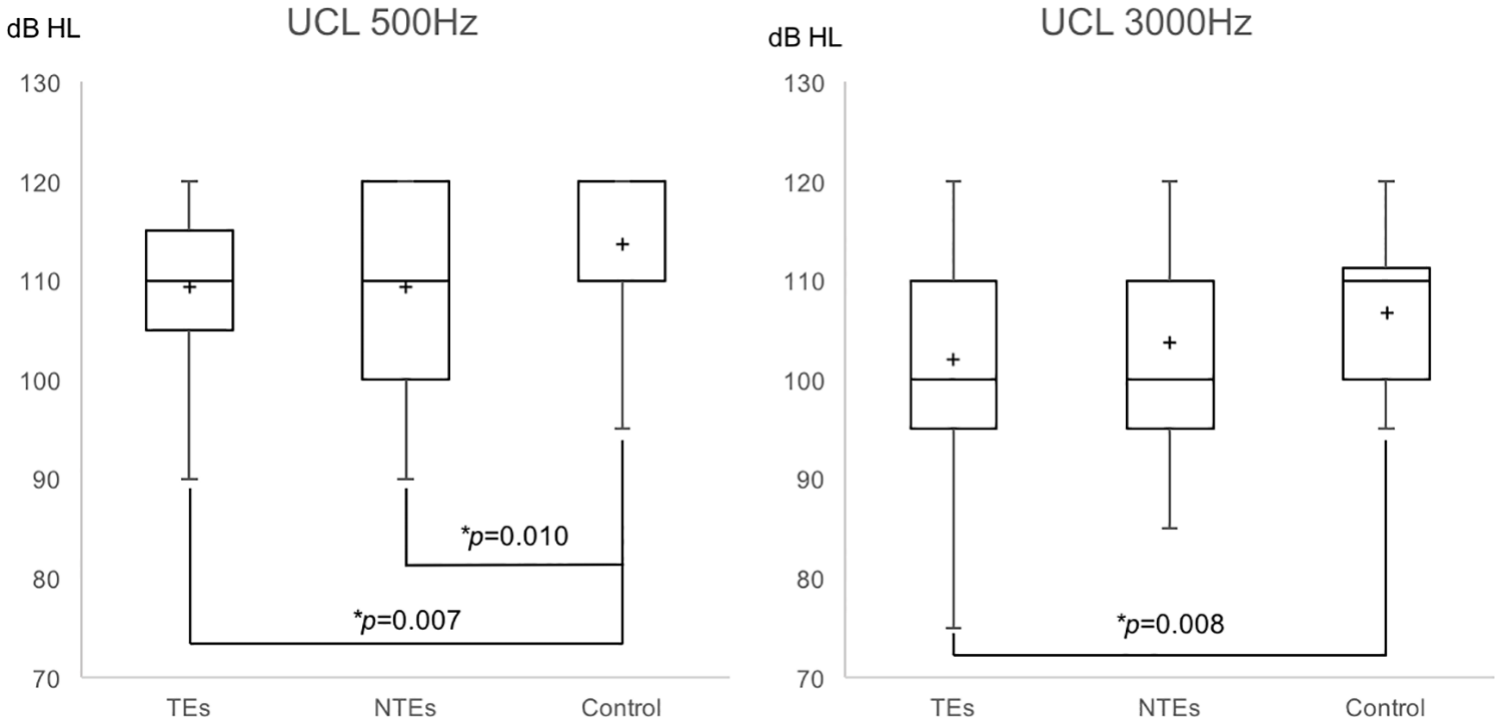
Comparisons of UCLs at 500 Hz and 3000 Hz among the TEs, NTEs, and control ears. UCL, uncomfortable loudness level, * *p* < 0.017.

The within-subject comparison between TEs and NTEs in all tinnitus subjects (males and females combined) revealed no significant differences in UCLs at 500 Hz (TEs, 109.66 ± 8.45 vs. NTEs, 109.09 ± 9.78) and 3000 Hz (TEs, 102.05 ± 9.36 vs. NTEs, 103.30 ± 9.94; Wilcoxon test, *p* > 0.05). Multiple comparisons with the Bonferroni correction revealed that TEs or NTEs in subjects with tinnitus showed significantly lower UCLs at 500 Hz compared with control ears (TEs, 109.66 ± 8.45 vs. NTEs, 109.09 ± 9.78 vs. controls, 114.44 ± 7.25; Mann-Whitney test with the Bonferoni correction, *p* = 0.007 or 0.010; α = 0.05/3, Fig 3). We also found a lower UCL at 3000 Hz for TEs in subjects with l tinnitus ear, compared with control ears (TEs, 102.05 ± 9.36 vs. NTEs, 103.30 ± 9.94 vs. controls, 107.92 ± 8.23; independent *t* test with the Bonferoni correction, *p* = 0.008; α = 0.05/3, Fig 3). Independent *t* test between NTEs and control ears revealed borderline significane (*p* = 0.019).

### Individual data

**Fig 4:**
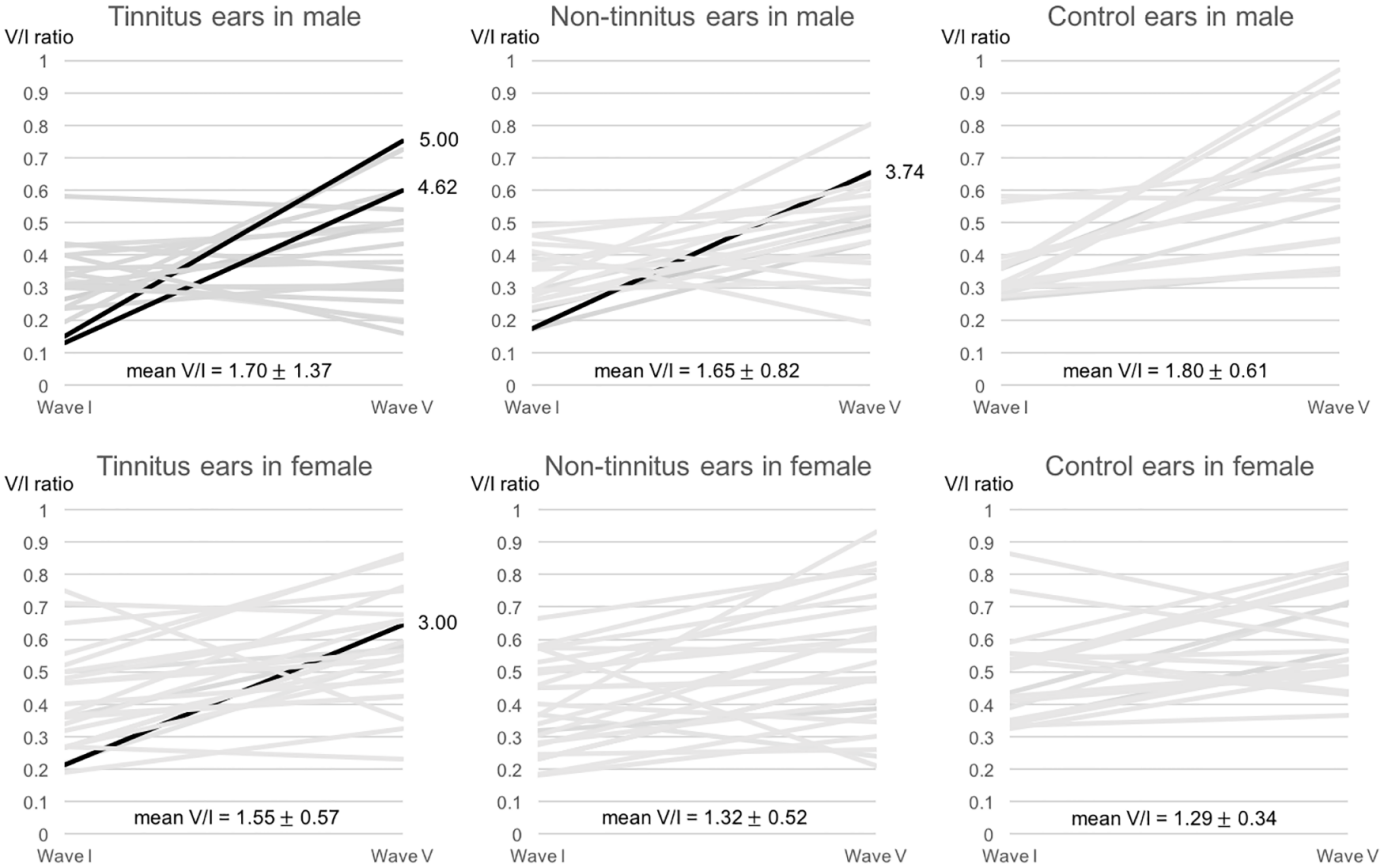
Wave V/I amplitude ratios. We found increased V/I amplitude ratios > mean + 2 SD in 2 TEs (V/I amplitude ratios; 4.62 and 5.00) in male tinnitus subjects and one TE (V/I amplitude ratio; 3.00) in female tinnitus subjects. Meanwhile, only one NTE in the male tinnitus subjects showed increased V/I amplitude ratios (3.74) > mean + 2 SD, and none of the NTEs in the group of female subjects with tinnitus showed increased V/I amplitude ratios. For the control ears, we found no ears with increased V/I amplitude ratios > mean + 2 SD, regardless of sex.

Individual data analysis revealed increased V/I amplitude ratios > mean + 2 SD (> 4.52) in two TEs among the 19 male subjects (Fig 4 and S1 Table). They had the 1^st^ and 2^nd^ smallest wave I amplitude of all the TEs in the male tinnitus group. Specifically, a TE in subject No. 37 had the smallest wave I amplitude (0.13 μV) and a V/I amplitude ratio of 4.62, with a wave V amplitude that was higher (0.60 μV) than the average wave V amplitude (0.44 μV ± 0.19). A TE in subject No. 29 had the 2^nd^ lowest wave I amplitude (0.15 μV), a V/I amplitude ratio of 5.00, and a wave V amplitude that was higher (0.75 μV) than the average wave V amplitude (0.44 μV ± 0.19). Meanwhile, only one NTE in subject No. 19 in the male tinnitus group showed increased V/I amplitude ratios (3.74) > mean + 2 SD (3.29) (Fig 4 and S1 Table).

In 24 female subjects with tinnitus, we only found one TE with increased V/I amplitude ratios > mean + 2SD (> 2.69) (Fig 4 and S1 Table). The TE had the 2^nd^ lowest wave I amplitude (0.22 μV) among all the TEs in the female tinnitus group, but also had an increased wave V amplitude (0.65 μV), producing an extraordinarily large V/I amplitude ratio (3.00). None of the NTEs in the female subjects had increased V/I amplitude ratios > mean + 2 SD.

We found no control ears with increased V/I amplitude ratios > mean + 2 SD, regardless of sex. UCLs for 500 Hz and 3000 Hz stimuli for the TEs and NTEs with increased V/I amplitude ratios > mean + 2 SD were not significantly lower than the mean values (S1 Table).

Fifteen subjects showed UCL at 3000 Hz < 100 dB HL in either TEs or NTEs and among them 9 subjects revealed low UCLs in both ears. Six subjects showed UCL at 500 Hz < 100 dB HL in either TEs or NTEs, and among them 2 subjects revealed bilaterally low UCLs (S2 Data).

### Correlation analyses

We found no significant correlations between wave I and V amplitudes in TEs, NTEs, or control ears in the male or female tinnitus groups, with the exception of a weak correlation in NTEs in females with tinnitus (*r* = 0.486, *p* = 0.016, S2 Fig).

For 43 TEs in all subjects (males and females combined) with tinnitus, the wave V/I amplitude ratio did not correlate with UCLs for 500 Hz and 3000 Hz stimuli (500 Hz: *r* = 0.057, *p* = 0.073, 3000 Hz: -0.119, *p* = 0.046, S3 Fig). We found no correlation between the wave I amplitude and UCL for both the 500 Hz and 3000 Hz stimuli (500 Hz: *r* = -0.020, *p* = 0.903, 3000 Hz: *r* = -0.116, *p* = 0.477, S3 Fig).

## Discussion

Wave latencies of ABRs reflect neural conduction time, and are known to be relatively consistent among subjects with the same hearing level. Meanwhile, wave amplitudes of ABRs are determined by the amount of neural output, and can vary with age, sex, skull thickness, head size, and skin impedance, among other factors. The speed of the cochlear response time is based on individual mechanical properties of the basilar membrane. The faster the cochlear response time, the greater the neural synchronization of the cochlear output [12]. Don et al. [13] showed that cochlear response times are sex specific: they are 13% shorter in females vs. males. This finding could explain the larger amplitudes of ABR waveform in the females in this study. However, within each sex, amplitude varies more between subjects than within subjects [11]. In the current study, we used within-subject comparisons to exclude the effects of individual variation, and attempted subgroup comparisons with age-matched controls according to sex. To reduce technical variations, we maintained the stimuli at a constant nHL level of 90 dB, used the same number of stimuli for each participant (1500), and ensured that there were zero artifacts in the recordings.

“Hidden hearing loss” is a condition in which damage to the auditory system is not sufficient to produce a threshold shift, or the auditory system partially recovers to the extent to which thresholds are restored to original levels, despite residual physical damage. Selective loss of high-threshold auditory nerve fibers or synaptopathy could occur without alternations to the normal hearing threshold due to the presence of intact low-threshold fibers, despite increased difficulty in processing suprathreshold sound [4,5]. Thus, central gain in the brainstem may increase to compensate for the reduced neural output from the cochlea, especially in high-threshold auditory nerve fibers. This has been suggested as a potential mechanism of tinnitus in patients with normal audiograms. Indeed, this mechanism seems to correspond to the observed catch-up growth of wave V potential as a response to the decreased amplitude of wave I potential [2,3,14]. The authors of a recent study hypothesized that selective loss of high-threshold auditory nerve fibers should correlate negatively with the delayed onset response of wave V ABR with masking noise because high-threshold auditory nerve fibers are more resistant to background noise [15].

In the current study, we found an extraordinarily large V/I amplitude ratio in three TEs (from two male subjects and one female subject) among a total of 43 unilateral tinnitus subjects. Data from these three cases implies an increase in central gain with a decreased wave I amplitude, but an excessively high wave V amplitude. However, we could not find meaningful evidence to support the “hidden hearing loss” hypothesis with respect to the mean comparisons between TEs and NTEs in individual subjects in both the male and female tinnitus groups. Moreover, comparison of wave I vs. V amplitudes did not reveal a consistent difference. Thus, although “hidden hearing loss” with cochlear synaptopathy might contribute to tinnitus in some cases, other mechanisms are likely to play an important role in tinnitus in patients with normal hearing thresholds.

There are several possible explanations for the differences between our results and those of previous animal studies [1,4,14]. First, in animal studies, the temporary threshold shift (“hidden hearing loss”) model is based entirely on acoustic overexposure, and tinnitus is not measured. In the current study, we enrolled unilateral tinnitus subjects with normal audiograms. For the majority of our patients, their tinnitus had an unknown origin. Thus, tinnitus etiology in our sample was likely heterogeneous. Second, unlike in mice, the human ABR wave I is not prominent, making precise measurement difficult. Third, although the cochlear synaptopathy with high-threshold auditory nerve fibers found in animal models could be one trigger factor in the development of tinnitus, such as hair cell damage, tinnitus might be determined by more complicated mechanisms at the level of central auditory system. The findings of our previous study in which we conducted a psychoacoustic comparison between TEs and NTEs in human subjects with tinnitus and normal and symmetric hearing thresholds support this hypothesis [16]. Specifically, we found that speech perception ability in noise was decreased in TEs compared with NTEs, despite no influence of tinnitus upon auditory spectral resolution associated with outer hair cell status.

Contrary to our result, several human studies with tinnitus patients with normal audiograms reported small wave I amplitudes [2,3]. There are several possible reasons for this discrepancy. First, we used within-subject comparisons (TEs and NTEs in the same subject) and multiple comparisons including TEs, NTEs, and control ears, whereas they used between-subjects comparisons (tinnitus group vs. control group). We believe that within-subjects comparisons are more reliable for measuring ABR amplitude, as this variable can vary widely among individuals. Second, previous human studies did not describe the laterality of tinnitus (right or left or both) or state whether they assessed ABR on the same or different side of the head with respect to tinnitus. Third, the sample sizes were much bigger in the current study.

In the active loudness model by Zeng FG [17], decreased signal input induces an increase in the nonlinear gain, producing enhanced loudness (recruitment or hyperacusis). This enhanced loudness introduces an imbalanced state in the brain, requiring an increase in central noise levels to maintain equilibrium, thus generating tinnitus. In other words, hyperacusis with increased nonlinear gain (steeper input output function) induces a reduction in the output dynamic range by raising the floor, thus increasing the central noise level (i.e. generation of tinnitus). This model easily explains the simultaneous occurrence of tinnitus and hyperacusis, of which an increase in central gain could be a common cause. Based on the active loudness model, we hypothesized that increased central gain associated with compensation for reduced neural output from the cochlea in tinnitus subjects with normal audiograms would be correlated with decreased UCLs, regardless of the subjective perception of hyperacusis. In the current study, we found no significant decrease in UCLs in TEs compared with NTEs, and although we found three TEs with an increased wave V/I amplitude ratio, the ears were not accompanied by a specific decrease in UCLs. Our correlation analysis showed no meaningful correlations between UCLs and wave I amplitude or wave V/I amplitude ratio. This was in good agreement with the investigation by Gu et al. [3]. Nevertheless, we observed lower UCLs in both TEs and NTEs compared with control ears. A similar finding was reported in a recent previous study that demonstrated an average UCL value that was 11.3 dB lower in adolescents with normal hearing and tinnitus (or former tinnitus) compared with adolescents with normal hearing and no tinnitus [18]. Thus, decreased UCLs in tinnitus patients could be associated with unknown personal factors associated to tinnitus vulnerability rather than tinnitus itself. Alternatively, unilateral tinnitus might affect susceptibility to loud sounds in both sides. Munro et al. [19] found that loudness perception decreased by 7 dB in both ears even though only one ear was occluded for 7 days. They suggested that the equivalent loudness perception between the occluded and unoccluded ear could be explained by the bilateral and complementary change of the acoustic reflex; decreased reflex threshold in the occluded ear but increased reflex threshold in unoccluded ear [20]. Darrow et al. [20] provided evidence that lateral olivocochlear efferents balance cochlear nerve output between the two ears, presumably to preserve accurate sound localization based on interaural level differences. Applied to the present results we could suggest that hidden hearing loss might affect loudness perception not only in the damaged ear but also in contralateral ear to maintain equivalent loudness judgement. One may argue that synaptopathy has not been expressed in ABRs specific to TEs, owing to the role of lateral olivocochlear efferents, which could balance cochlear nerve activity between the ears. However, unlike the UCL data, there were no significant differences in the amplitudes of wave I among the TEs, NTEs, and control ears. To investigate whether synaptopathy in the TEs is masked by lateral olivocochlear efferents, further ABR studies will be needed with subjects having bilateral tinnitus and non-tinnitus controls.

Tinnitus may be multifactorial in origin, such that various mechanisms could operate to induce tinnitus in subjects with normal hearing thresholds. However, increased central gain as compensation for decreased input signal exclusively in synapses or the auditory nerve remains a possible mechanism in the development of tinnitus.

## Conclusions

In contrast to previous studies, our ABR data do not represent meaningful evidence supporting the hypothesis of “hidden hearing loss”, although several TEs showed extremely high V/I amplitude ratios implying increased central gains. Although cochlear synaptopathy with increased central gain might not be a common mechanism of tinnitus in subjects with normal audiograms, it may contribute to tinnitus in some cases. However, reduced sound level tolerance in both TEs and NTEs might reflect increased central gain consequent on hidden synaptopathy that was subsequently balanced between the ears by lateral olivocochlear efferents. Reduced UCL might be a potential indicator of “hidden hearing loss” in tinnitus subjects though it is difficult to indicate the side of the lesion.

## Supporting information

S1 Fig ABR setup and measurement technique of the amplitudes of wave I and V. (PPTX)

S2 Fig Scatter plot of wave V amplitude against wave I amplitude for TEs, NTEs, and control ears in each male and female subgroup. (TIFF)

S3 Fig Scatter plot of uncomfortable loudness level (UCL) against the wave V/I amplitude ratio (A) and UCL against the wave I amplitude for 43 TEs in all subjects with tinnitus (B). (TIFF)

S1 Table Individual data for wave amplitudes and uncomfortable loudness levels for ears in which increased wave V/I amplitude ratios > mean + 2 SD. (DOCX)

S1 Data Raw data of ABR. (XLSX)

S2 Data Raw data of uncomfortable loudness level. (XLSX)
