# Supplementary material for: Comparisons of auditory brainstem response and sound level tolerance in tinnitus ears and non-tinnitus ears in unilateral tinnitus patients with normal audiograms
Source: PLoS One. 2017 Dec 18;12(12):e0189157. doi: 10.1371/journal.pone.0189157 (PMC5734686; doi:10.1371/journal.pone.0189157)
Supplement: S1 Fig — (PPTX) [file pone.0189157.s001.pptx]

## Slide 1
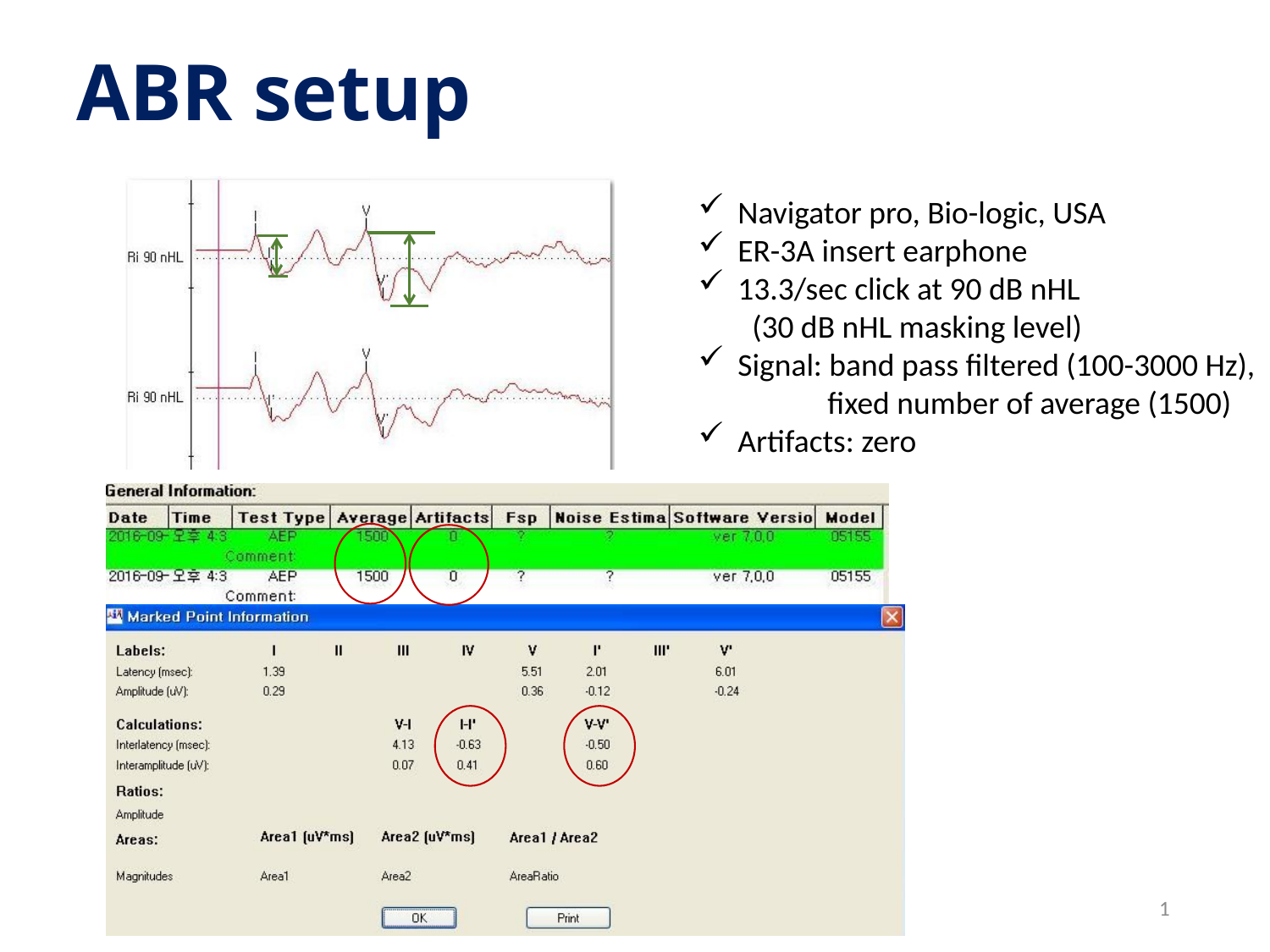

# ABR setup
Navigator pro, Bio-logic, USA
ER-3A insert earphone
13.3/sec click at 90 dB nHL (30 dB nHL masking level)
Signal: band pass filtered (100-3000 Hz),
 fixed number of average (1500)
Artifacts: zero
1
