# Supplementary figures and images for: Comparisons of auditory brainstem response and sound level tolerance in tinnitus ears and non-tinnitus ears in unilateral tinnitus patients with normal audiograms
Source: PLoS One. 2017 Dec 18;12(12):e0189157. doi: 10.1371/journal.pone.0189157 (PMC5734686; doi:10.1371/journal.pone.0189157)

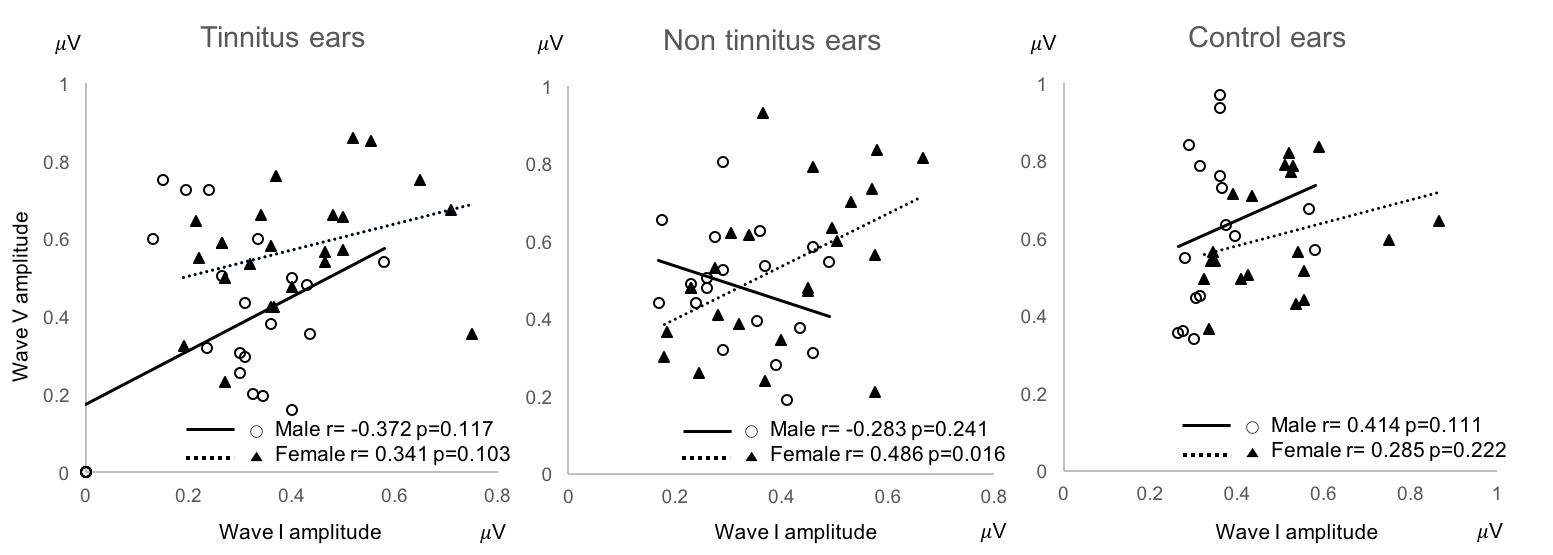

Supplement: S2 Fig — (TIFF) [file pone.0189157.s002.tiff]

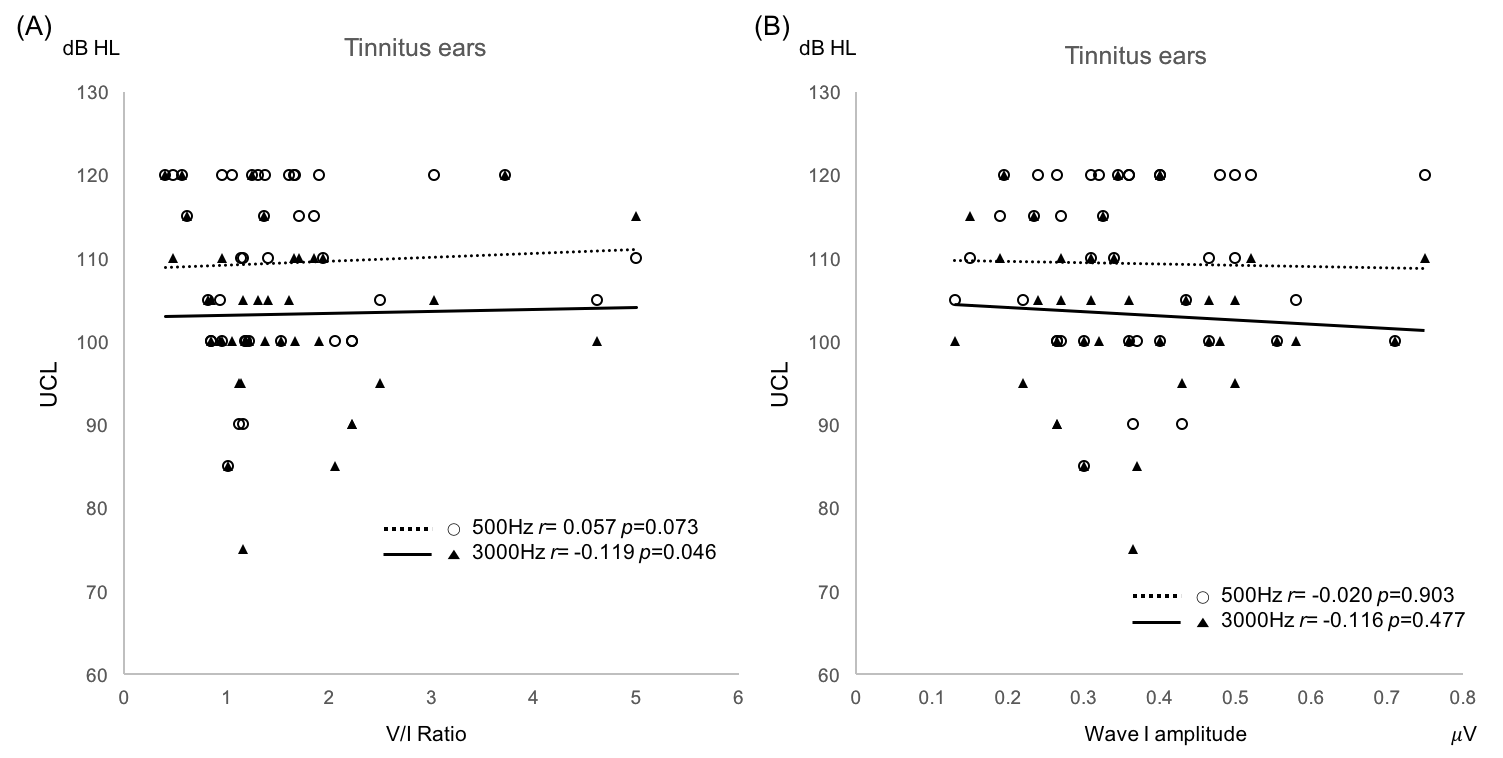

Supplement: S3 Fig — (TIFF) [file pone.0189157.s003.tiff]
