# Supplementary material for: Comparisons of auditory brainstem response and sound level tolerance in tinnitus ears and non-tinnitus ears in unilateral tinnitus patients with normal audiograms
Source: PLoS One. 2017 Dec 18;12(12):e0189157. doi: 10.1371/journal.pone.0189157 (PMC5734686; doi:10.1371/journal.pone.0189157)
Supplement: S1 Table — (DOCX) [file pone.0189157.s004.docx]

**S1 Table Individual data for wave amplitudes and uncomfortable loudness levels for ears in which increased wave V/I amplitude ratios > mean + 2 SD**

|  |  | Male tinnitus subjects | | | | |
| --- | --- | --- | --- | --- | --- | --- |
|  |  | Wave I amplitude  (μV,  mean ± SD) | Wave V amplitude  (μV ,  mean ± SD) | Wave V/I  amplitude ratios  (mean ± SD) | UCL in  500 Hz  (dB HL,  mean ± SD) | UCL in  3000 Hz  (dB HL,  mean ± SD) |
| Subject No. 19 | NTE | 0.18  (0.33 ± 0.10) | 0.66  (0.48 ± 0.15) | 3.74  (1.65±0.82) | 115  (109.09 ± 9.78) | 120  (103.30 ± 9.94) |
| Subject No. 29 | TE | 0.15  (0.32 ± 0.11) | 0.75  (0.44 ± 0.19) | 5.00  (1.70 ± 1.37) | 110  (109.66 ± 8.45) | 115  (102.05 ± 9.36) |
| Subject No. 37 | TE | 0.13  (0.32 ± 0.11) | 0.60  (0.44 ± 0.19) | 4.62  (1.70 ± 1.37) | 105  (109.66 ± 8.45) | 100  (102.045 ± 9.358) |
|  |  | Female tinnitus subjects | | | | |
|  |  | Wave I amplitude  (mean ± SD) | Wave V amplitude  (mean ± SD) | Wave V/I  amplitude ratios  (mean ± SD) | UCL in  500 Hz  (mean ± SD) | UCL in  3000 Hz  (mean ± SD) |
| Subject No. 15 | TE | 0.22  (0.41 ± 0.15) | 0.65  (0.57 ± 0.15) | 3.000  (1.55±0.57) | -  - | -  - |
